# Trends in cardiovascular disease among Inuit in Greenland from 1994 to 2021

**DOI:** 10.1016/j.athplu.2024.04.002

**Published:** 2024-04-25

**Authors:** Hjalte Erichsen Larsen, Uka Wilhjelm Geisler, Finn Gustafsson, Michael Lynge Pedersen, Marit Eika Jørgensen

**Affiliations:** a Greenland Center for Health Research, Institute of Health and Nature, University of Greenland, Nuuk, Greenland; b Medical Department, Queen Ingrid's Hospital, Nuuk, Greenland; c Department of Cardiology, Rigshospitalet, Copenhagen, Denmark; d Steno Diabetes Center Greenland, Nuuk, Greenland; e University of Southern Denmark, Odense, Denmark

## Abstract

**Background and aims:**

Cardiovascular disease (CVD) poses significant health challenges globally. While substantial data exists for most populations, the Arctic Inuit's CVD incidence rates remain understudied. This research aimed to change this by estimating CVD incidence and mortality rates in Greenland from 1994 to 2021.

**Methods:**

Using nationwide registers, a retrospective observational study was conducted, focusing on individuals born in Greenland to Greenlandic-born parents. Data were sourced from the Greenlandic Hospital Discharge Register and the nationwide electronic medical record.

**Results:**

A total of 65,824 individuals were included. the age- and sex-specific incidence rates (IR) of ischemic heart disease, stroke, and heart failure (HF) declined from 1994 to 2021, with the most substantial decline observed for HF among women. Conversely, the IR of atrial fibrillation/flutter increased in both men and women, while the IR of myocardial infarction rose among men. The IR for stroke was particularly elevated compared to other CVD subgroups. Mortality rates for those diagnosed with CVD were 2.4 times higher than those without. Men exhibited a 40 % elevated mortality risk relative to women.

**Conclusion:**

The study provides pivotal insights into CVD trends within the Arctic Inuit population, highlighting both positive developments and areas of concern. Given the increasing elderly demographic in Greenland, proactive health strategies are crucial. Emphasizing primary prevention and addressing specific CVD risks, particularly the elevated stroke IR, is imperative for future public health efforts.

## 1 Introduction

Cardiovascular disease (CVD) is the leading cause of death globally, with ischemic heart disease (IHD) and stroke as the primary contributors [1]. Through the second half of the 20th century, mortality from CVD has declined, especially in high-income Western countries, recent trends indicate a slowing in the decline, potentially driven by rising obesity and diabetes rates [2]. The decrease in CVD-related deaths can be credited to improved, evidence-based treatment and a decline in risk factors [2,3]. However, when it comes to the indigenous populations of the Arctic, comprehensive data on CVD is limited. Notably, there is a high prevalence of IHD and stroke among Canadian Inuit, and Alaskan natives [4,5]. Home to 56,609 inhabitants, Greenland is the world's largest island. The majority, 82 %, are Inuit when defined as being born in Greenland to Greenland-born parents. Inuit in Greenland shares historical roots with Inuit populations in northern Canada, Alaska, and Siberia. From 1994 to 2021, there was a modest population increase from 55,419 to 56,421 in Greenland [6]. Every year, nearly 10 % of the population migrates to or from Greenland, primarily to or from Denmark [7]. Over the past five decades, people have moved from the smaller settlements to the larger cities. Currently, approximately 60 % of the population lives in the five major cities, Nuuk, Sisimiut, Ilulissat, Aasiaat, and Qaqortoq. Additionally, the number of elderly individuals (those above 50) has seen a significant increase from 1994 to 2021, rising from 7440 to 15,666. Correspondingly, life expectancy has shown an upward trend from 1999 to 2021, with increases from 68.7 to 74.0 years for women and from 62.5 to 69.2 years for men [6]. Historically, the prevalence and nature of CVD in Greenland have been subject to different notions. In 1940, Dr. Albert Berthelsen, who worked in Greenland for several years, reported frequent cases of atherosclerosis among the Inuit [8]. Contrarily, in the 1970s, Danish researchers, who examined the composition of the Inuit food, reported, that the incidence of myocardial infarction was low in Greenland [[9], [10], [11], [12]]. This perspective, however, was challenged by a comprehensive review in 2003, which concluded that the described low mortality rates were derived from unreliable data. They further concluded that stroke mortality probably was higher than in other populations [13]. Concurring with this, a 2014 review concluded that the prevalence of coronary artery disease in Inuit populations is similar to other populations, and that strokemortality is high [14]. More recent studies further support the notion that IHD rates among Inuit mirror those in other Western societies [[15], [16], [17], [18]]. Furthermore, genetic analyses have identified genetic variants in the Greenlandic Inuit that increase the risk of diabetes, obesity, familial hypercholesterolemia, and CVD [[19], [20], [21]]. The traditional Inuit lifestyle has gradually been westernized over the past century. Consequently, Greenland has experienced a rise in obesity rates from 13 % in 2003 to 27 % in 2018, defined by a body mass index (BMI) exceeding 30 kg/m^2^ [22]. Conversely, the proportion of daily smokers has shown a decline from 68 % in 1993 to 52 % in 2018 [22]. By 2021, 17.5 % of those aged 20 and above were on antihypertensive medications [23]. Factors such as smoking, alcohol consumption, poor diet, and physical inactivity amplify CVD risks, with research suggesting that an optimal lifestyle could prevent nearly 80 % of IHD events [[24], [25], [26]]. Clinical risk determinants for CVD include conditions like hypertension, dyslipidemia, diabetes, and obesity. Moreover, psychosocial factors, including depression, stress, and major life events, exhibit a significant correlation with IHD [27]. Given the data disparities, emerging risk factors, and lack of contemporary research on CVD incidence, this study aims to estimate the incidence and mortality of CVD in Greenland between 1994 and 2021, using data from the Greenlandic Hospital Discharge Register (GHDR) and the nationwide electronic medical record (EMR).

## 2 Materials and methods

### 2.1 Design and setting

We conducted a retrospective observational study using data from the GHDR and EMR databases in Greenland. In Greenland, health care is provided by healthcare clinics in the settlements, healthcare centers in the towns, hospitals in the larger towns, and the national hospital, Queen Ingrid's Hospital (QIH) in Nuuk. Discharge diagnoses on all patients admitted to hospitals or healthcare centers have since 1987 been registered in the GHDR. CVD diagnoses in the GHDR have been validated and are suitable for epidemiological use [28]. The present EMR was implemented from 2013 to 2017, and all diagnoses given to patients admitted and in outpatient care are registered here. Diagnoses are recorded according to the International Classification of Disease 8th revision and 10th revision [29,30], and primary care diagnoses are recorded according to the International Classification of Primary Care [31].

### 2.2 Study population

People born in Greenland to parents who were also born in Greenland, and who resided there between January 1st, 1994 to December 31st, 2020, were included in the cohort. Entry date was the date of birth or date of migration to Greenland, exit date was the date of death and date of migration from Greenland or December 31st, 2020. Those who migrated from Greenland were excluded from the cohort at the time of migration but were reincluded if/when returning to Greenland. The time lived outside Greenland was censored. Patients diagnosed with CVD (*See Appendix*) in the GHDR or the EMR were identified and included as incident CVD. Data were linked using the unique Civil Registration Number.

### 2.3 Statistics

All statistical calculations were made using the open-source software, RStudio (version 4.3.0). Before analysis, data were anonymized to ensure the privacy and security of the patient information. CVD diagnoses were divided into the subgroup categories; IHD and myocardial infarction (MI), stroke, heart failure (HF), and atrial fibrillation/flutter (AF/AFL) (*See Appendix*). When more than one diagnosis was recorded per individual in a CVD subgroup, only the earliest diagnosis was used. The date of CVD event was recorded as the date of diagnosis. Separate cohorts were established based on CVD subgroup and sex, and for each of these, a lexis object containing follow-up in multiple states and timelines was created [32]. A new state (CVD) was created for those with a registered diagnosis, from the time of diagnosis. The lexis object was then split on the age timescale into 3-month periods. A Poisson regression model with the transition rate to the CVD state (incidence) as outcome and age and time (year) as predicted variables were made for each sex and CVD subgroup. Incidence rate (IR) per 100,000 person-years (PY) was predicted using the models for those aged 40, 50, 60, 70, and 80 years. Age at diagnosis was calculated as the median with interquartile range (IQR). Mortality rates of CVD were predicted by a similar model but based on the transition to death. The cohort included men and women in all CVD subgroups, again only the earliest diagnosis was used when more than one diagnosis was registered per individual. A Poisson regression model was made with the transition rate to death (mortality) as outcome and sex, age, time (year), and state (CVD or no CVD) as predicted variables. Hazard ratios comparing sexes and CVD statuses were derived from this model. For visualization, mortality rates per 1000 person-years were predicted for men and women, with and without CVD, and between 30 and 90 years of age in 2010. The total unadjusted crude incidence of the different CVD was predicted using a Poisson regression model with the transition to CVD as outcome and time (year) as the predictor variable. For all models used, deviation from linearity was assessed by including the quadratic, cube, and common logarithm of the time variable in the Poisson regression model. When no deviation from linearity was found results were interpreted as linear, when deviation from linearity was found the time variable was splined into 14-year periods.

### 2.4 Ethics

The study was approved by the Greenlandic Science Ethics Committee and the Greenlandic Healthcare Administration. It was conducted according to the Helsinki Declaration, all data from the EMR was anonymized and no informed consent from participants was needed.

## 3 Results

A total of 65,824 people were included in the study. The mean follow-up time was 18.9 years, resulting in a total of 1,242,053.5 PY. The cohort-specific total risk time, mean follow-up time, events, and age at diagnosis are presented in Table 1.

**Table 1:** Descriptive data according to cohort and gender. SD = standard deviation, IQR = interquartile range, n = number, IHD = ischemic heart disease.

IHD incidence rate	Men (n = 33434)	Women (n = 32352)
Total risk time (years)	626373.9	604452.3
Mean follow up time (years)	18.7	18.7
Events (n)	965	557
Total incidence rate (events/100.000 person-year (95 % CI))	154.1 (144.3–163.8)	92.1 (84.5–99.8)
Age at diagnosis (years, median [IQR])	61.4 [53.6–69.1]	64.2 [55.2–72.1]

**Myocardial infarction incidence rate**	Men (n = 33451)	Women (n = 32364)
Total risk time (years)	631521.1	607639.3
Mean follow up time (years)	18.9	18.8
Events (n)	349	144
Total incidence rate (events/100.000 person-year (95 % CI))	55.3 (49.5–55.3)	23.7 (19.8–27.6)
Age at diagnosis (years, median [IQR])	61.2 [53.5–69.0]	66.3 [57.5–72.4]

**Stroke incidence rate**	Men (n = 33384)	Women (n = 32300)
Total risk time (years)	623790.5	598996.4
Mean follow up time (years)	18.7	18.5
Events (n)	1412	1325
Total incidence rate (events/100.000 person-year (95 % CI))	226.4 (214.6–238.2)	221.2 (209.3–233.1)
Age at diagnosis (years, median [IQR])	62.2 [53.6–69.5)	62.6 [51.1–73.1]

**Heart failure incidence rate**	Men (n = 33440)	Women (n = 32328)
Total risk time (years)	629955.7	605764.8
Mean follow up time (years)	18.8	18.7
Events (n)	802	573
Total incidence rate (events/100.000 person-year (95 % CI))	127.3 (118.5–136.1)	94.6 (86.8–102.3)
Age at diagnosis (years, median [IQR])	66.8 [57.9–74.1]	70.9 [62.5–78.7)

**Atrial fibrillation/flutter incidence rate**	Men (n = 33444)	Women (n = 32353)
Total risk time (years)	630010.0	606281.2
Mean follow up time (years)	18.8	18.7
Events (n)	524	331
Total incidence rate (events/100.000 person-year (95 % CI))	83.2 (76.1–90.3)	54.6 (48.7–60.5)
Age at diagnosis (years, median [IQR])	61.7 [54.0–69.8]	69.6 [60.7–77.1]

**Mortality rate:**	n = 65824	
Total risk time (years)	1242053.5	
Mean follow up time (years)	18.9	
Deaths (n)	11623	
Age at death (years, median [IQR])	66.5 [52.3–76.1]	

### 3.1 Subgroup-specific incidence rates

**Fig. 1:**
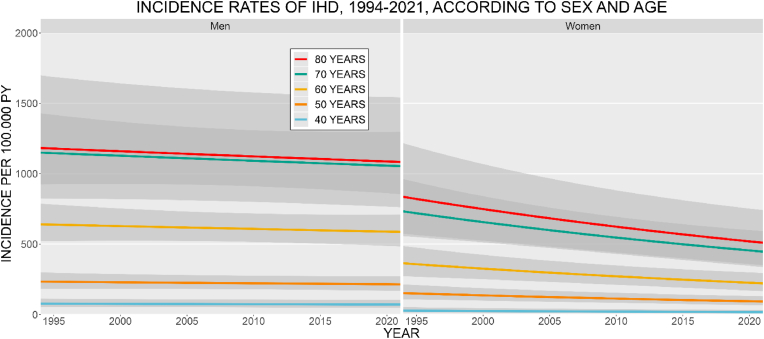
Incidence rate of ischemic heart disease (IHD) per 100,000 person-years between 1994 and 2021, according to sex at age 40, 50, 60, 70, and 80 years. The shaded area shows 95 % confidence interval. PY = person-years.

**Fig. 2:**
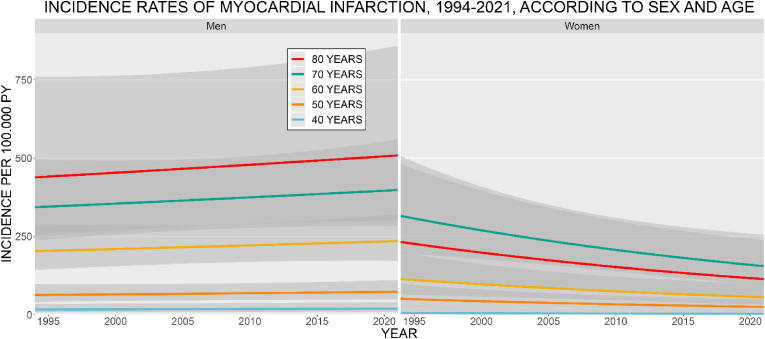
Incidence rate of myocardial infarction per 100,000 person-years between 1994 and 2021, according to sex at age 40, 50, 60, 70, and 80 years. The shaded area shows 95 % confidence interval. PY = person-years.

**Fig. 3:**
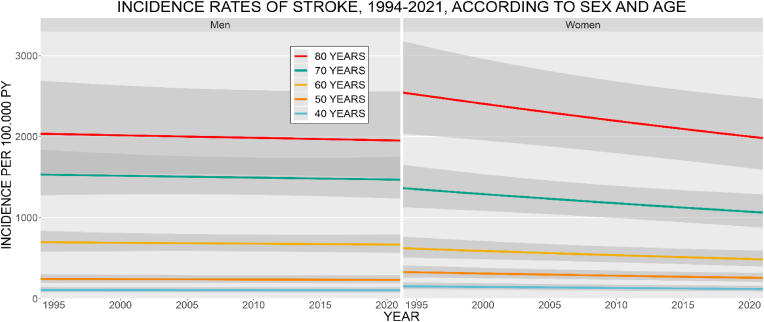
Incidence rate of stroke per 100,000 person-years between 1994 and 2021, according to sex at age 40, 50, 60, 70, and 80 years. The shaded area shows 95 % confidence interval. PY = person-years.

**Fig. 4:**
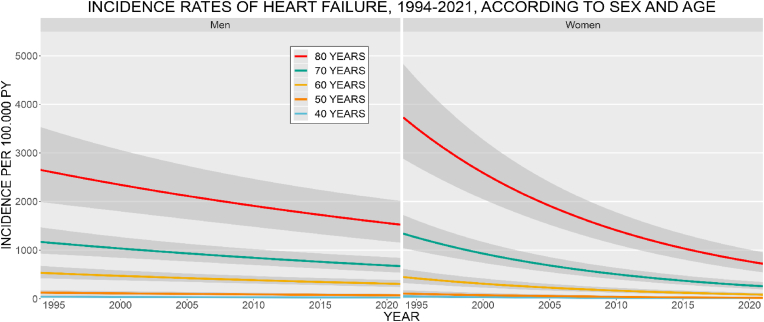
Incidence rate of heart failure (HF) per 100,000 person-years between 1994 and 2021, according to sex at age 40, 50, 60, 70, and 80 years. The shaded area shows 95 % confidence interval. PY = person-years.

**Fig. 5:**
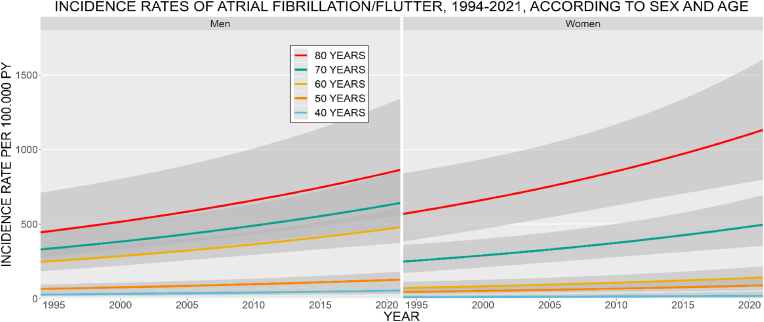
Incidence rate of atrial fibrillation/flutter per 100,000 person-years between 1994 and 2021, according to sex at age 40, 50, 60, 70, and 80 years. The shaded area shows 95 % confidence interval. PY = person-years.

*IHD and MI:* The age- and sex-specific IR of IHD decreased from 1994 to 2021 for both men and women, stronger among women (Fig. 1). Men had higher IR at all ages. The IR increased with age, for both sexes. In 2021, the IR of IHD at age 70 was 1053.7 (CI: 855.7–1297.5) for men and 446.9 (CI: 337.6–591.6) for women, per 100,000 PY. The IR of MI increased for men and decreased for women during the study period (Fig. 2). Men had the highest IR at all ages. The IR increased with age for men, among women the 70 years old had the highest IR. The IR at age 70, in 2021 was 397.9 (CI: 282.6–560.3) for men and 154.8 (CI: 93.9–255.0) for women, per 100,000 PY. *Stroke:* The age- and sex-specific IR of stroke decreased from 1994 to 2021 (Fig. 3). The decrease was more pronounced among women. Women had higher IR at ages 40, 50, and 80 years, the highest IR was among the 80 year old women. The IR increased with age, for both sexes. In 2021, the stroke IR, at 70 years of age was 1468.9 (CI: 1233.8–1748.8) for men and 1063.2 (CI: 876.6–1289.4) for women, per 100,000 PY. *Heart failure:* The age- and sex-specific IR of HF markedly decreased from 1994 to 2021 (Fig. 4). For women, the IR declined by a factor of 5 during the period. The IR for men decreased but was not as pronounced as for women. The highest IR was among the 80-year-old men and women. In 2021, the HF IR, at 70 years of age was 672.9 (CI: 536.7–843.6) for men and 258.9 (CI: 194.6–344.4) for women, per 100,000 PY. *Atrial fibrillation/flutter:* The age- and sex-specific IR of AF/AFL has increased among men and women (Fig. 5). The 80-year-old women had the highest IR, at all other ages, med had the highest IR. Men at 60 years of age had IR similar to that of the 70-year-old women. In 2021, the AF/AFL IR, at 70 years of age was 641.0 (CI: 480.4–855.4) for men and 495.5 (CI: 353.7–694.3) for women, per 100,000 PY.

*CVD mortality*

**Fig. 6:**
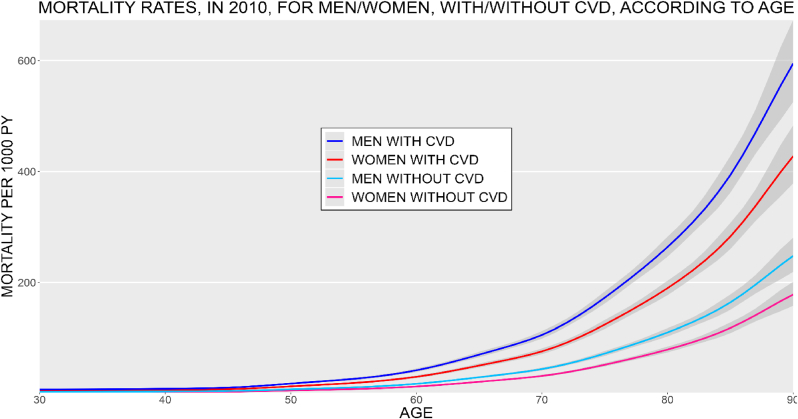
Mortality rate for men/women and with and without cardiovascular disease, based on mortality rates in 2010. The shaded area shows 95 % confidence interval. Mortality rate ratio for cardiovascular disease: 2.4, and for Male sex: 1.4. PY = person-years, CVD = cardiovascular disease.

The total number of deaths was 11,623, of which 2941 were among people diagnosed with CVD. The median age at death was 66.5 (IQR: 52.3–76.1). People diagnosed with CVD had a mortality rate-ratio of 2.4. The total mortality rate ratio was 1.4 for men compared to women. The mortality rates per 1000 PY, for men and women with and without CVD are presented in Fig. 6.

*The overall burden of CVD*

**Fig. 7:**
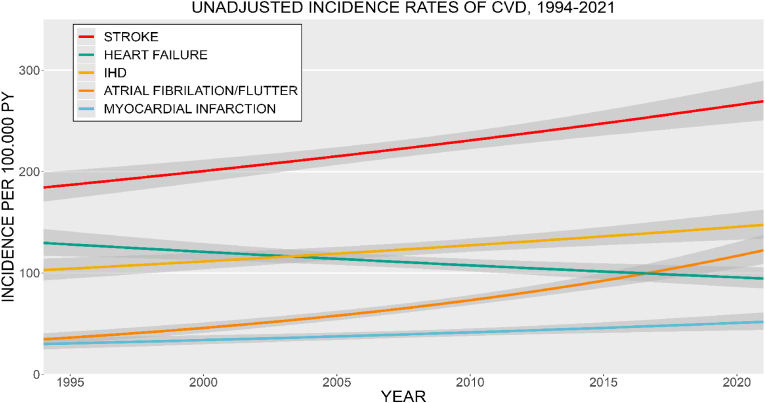
Unadjusted incidence rates of cardiovascular disease according to subgroup per 100,000 person years between 1994 and 2021. Shaded area shows 95 % confidence interval. PY = person years, CVD = cardiovascular disease, IHD = ischemic heart disease.

As a measure of the absolute burden of CVD on the healthcare system given the demographic changes, unadjusted rates were calculated. The unadjusted incidence rate of stroke, IHD, and AF/AFL have increased during the period. Stroke had the highest unadjusted incidence rate. The unadjusted incidence rate of HF declined during the study period. The unadjusted CVD incidence rates, per 100,000 PY, across CVD subgroups are presented in Fig. 7.

## 4 Discussion

This is the first study to show the incidence rates of CVD in the Arctic Inuit. The study found that the age- and sex-specific IR of IHD, stroke, and HF have declined during the period from 1994 to 2021. The biggest decline was seen for HF among women. The IR of AF/AFL and MI among men increased. Those diagnosed with CVD had 2.4 the mortality of persons not diagnosed with CVD. The IR of stroke was high compared to the other CVD subgroups. The unadjusted IR of stroke, AF/AFL, and IHD increased, while HF decreased. The median age at first diagnosis was low across all subgroups.

### 4.1 The age-specific incidence rates

*IHD and MI* The decreasing trend in the IR of IHD found in this study and the higher risk among men vs women in Greenland is comparable to that in most European countries [24]. The declining IR, globally as in Greenland, can be explained by a raised focus on prevention, particularly declining rates of smoking, and improved treatment possibilities for clinical risk factors such as dyslipidemia, hypertension, and diabetes [22,23,33]. The IR of IHD progressively increased with age for both men and women, however, the difference between the oldest groups (70 and 80 years old) is remarkably small. It can be hypothesized that older age groups may consist predominantly of ‘healthy survivors’ with individuals susceptible to IHD having already succumbed to the disease in earlier age groups. The age-specific IR across all age groups (40–80 years) found in 2021 is approximately 50 % lower than the global estimates from the Global Burden of Disease (GBD) study 2019 [34]. The low IR found in this study might reflect an actual lower incidence within the population. However, previous research has indicated that the incidence of IHD and associated markers are comparable to those in other Western populations [13,14,35]. Furthermore, data show that the rate of revascularization in Greenlandic citizens is as high as in Danish citizens [18]. A separate register study investigating the prevalence of AF in Greenland revealed that nearly half of the cases did not have a registered diagnosis of AF/AFL [36]. Consequently, it is plausible to consider that the actual IR of IHD in Greenland may be higher than our findings suggest, potentially due to a significant number of undiagnosed cases. MI was specifically categorized and further emphasized, as it is perceived as a more definitive diagnosis. Given the nature of the condition, MI is less likely to be overlooked and, therefore, underdiagnosed. As hypothesized, the IR of MI is more comparable to what is observed in the Danish population [37]. This supports the theory, that other IHD diagnoses might be underestimated, possibly due to subtler presentation. The ascending trend in the IR of MI among men diverges from other populations, where the incidence has generally declined [24,34,37]. The rising MI incidence among men reflects significant gender differences in cardiovascular health. Research shows higher rates of undiagnosed or untreated hypertension and more prevalent diabetes among men than women. Men also have a higher use of cannabis and alcohol and more men are classified as heavy smokers [22]. It could be suggested that the Greenlandic men are less prone to seek medical attention until severely ill. Such behaviour could contribute to the higher MI rates observed. The finding highlights the need for further investigation into gender-related differences in health practices and cardiovascular risk management. Notably, the highest IR among women was among those aged 70, rather than the expected 80 year old. This deviation could be attributed to various factors, including survivorship bias, where healthier individuals tend to live longer, thereby skewing the age distribution of disease prevalence. Another influencing factor could be that the elderly or those requiring more intensive healthcare, may choose to relocate to Denmark, taking advantage of the freedom of movement between Greenland and Denmark to access more readily available healthcare services. This migration could impact the observed disease prevalence and incidence rates. The age-specific trend might reflect unique healthcare access, lifestyle factors, or biological susceptibilities in this age group. Further investigation is needed to understand these dynamics fully.

*Stroke:* The IR of stroke found in this study is high, especially among the elderly. Compared to global estimates of stroke, the IR in Greenland is roughly 60 % higher on average when comparing the 40–80 year old [34]. The contribution from specific types of strokes, such as hemorrhagic and transient ischemic attacks, appears to be as expected and does not explain the high IR. Nevertheless, further investigation is needed, as some stroke diagnoses can be heterogeneous and might encompass various types of strokes. According to our findings, the IR of stroke is noticeably higher than that of IHD in Greenland. This is contrary to what is commonly seen in other populations, where the IHD incidence is almost twice that of stroke [24,38]. In Europe, the median age-standardized incidence estimates in 2019 were 293.3 for IHD and 135.5 for stroke [24]. The explanation for the high IR of stroke is not obvious as IHD and stroke share risk factors [39]. It could be that the prevalent risk factors in the Greenlandic population are more prone to cause stroke and less likely to result in IHD, different associations have been found between risk factors and CVD manifestations [40]. It could also be hypothesized that the high stroke incidence could be caused by a genetic disposition, this could be due to the unique genetic background of the Greenlandic Inuit, resulting from years of isolation and descent from a small ancestral population [41]. The decreasing trend in the IR of stroke found in Greenland during the study period is consistent with what is seen in most European countries. According to the European Society of Cardiology (ESC), the median age-standardized incidence estimates of stroke declined from 190.2, in 1990 to 135.2 per 100,000 PY in 2019 [24]. Similar decreasing trends are present for both the Global and Danish age-standardized IR of stroke according to the GBD study 2019 [34]. The decline in IR could, like the decrease in IHD IR, be explained by better prevention and improved treatment possibilities of clinical risk factors for stroke. Women had the highest IR among the youngest and the oldest, however, the IR decreased more among women than men during the study period, resulting in more comparable rates between the sexes by the end of the study in 2021. A similar difference in IR of stroke between the eldest men and women can be seen in the global estimated IR for stroke according to the GBD study 2019 [34]. According to their data, the oldest women (85+ years) have the highest IR [34]. Whether this is related to a real higher incidence among the older women or that older men have a higher competing risk leaving healthy survivors among the elderly, is unanswered. *HF:* We found a marked decline in HF IR in Greenland, especially among women. A decreasing incidence of HF is seen in other populations, however not as noticeably [42]. The IR of HF in Greenland seems to have declined from an alarmingly high level in the 1990s and early 2000s to a level more comparable to other populations. According to a Danish study including the entire adult Danish population from 1995 to 2012 the IR for those >74 years of age was about 120 per 10,000 PY in 2012 which is comparable to what we have found in the last part of the study period [43]. Hence, it could be speculated that the high IR found in the early study period is somehow unreliable. Breathlessness, ankle swelling, and fatigue are the characteristic symptoms of HF, however, such symptoms could be caused by other disease than HF [44]. The knowledge and diagnostic possibilities of HF have evolved since the ESC published its first guidelines for the diagnosis of HF in 1995 [45]. The high IR early in the study could be caused by misinterpreting symptoms as caused by HF, combined with limited diagnostic possibilities, leading to overdiagnosis of HF. Oppositely, the decreasing incidence could be explained by an elevated focus on HF with reduced ejection fraction, hence overlooking HF with preserved ejection fraction, which is found to constitute nearly half of all HF [46]. A reduction in rheumatic heart disease, which is known to cause HF, could also add to the decline in HF IR [47]. Although not presented in this study due to a low absolute number of diagnoses we see a distinct decline in rheumatic heart disease during the study period. The drastic decline in HF IR could also be explained by improved health care in general including primary prevention, easier access to health care for assessment, and treatment of common HF causes like hypertension and IHD [44]. *Atrial fibrillation/flutter:* The age-specific IR of AF/AFL increased during the study period, unlike what is seen globally and in Europe, where the IR has been stable [24,34]. Compared to the global IR, the age-specific IR is comparable at study start and high by the end of the study [34]. The IR of AF/AFL found in 2021 is roughly 85 % higher compared to global estimates of 2019 [48]. However, similar or lower when compared to what has been found in Denmark in 2018 [49]. As AF/AFL can be paroxysmal, diagnosis can be difficult. It could be that the increasing incidence is due to better diagnostics as Holter monitoring devices are now more commonly used. Furthermore, AF/AFL risk factors include comorbidities such as hypertension, diabetes, HF, IHD, and obesity [50]. Hence the increasing incidence of AF/AFL could be explained by an increase in the portion of people with these comorbidities. Globally AF/AFL is more common among men compared to women in all age groups [34]. Corresponding to what we found except among the oldest where women had the highest IR.

### 4.2 The total burden of CVD in Greenland

As life expectancy continues to increase, so does the proportion of elder people with a high incidence of CVD. The global increase in life expectancy correlates with rising crude CVD incidence rates worldwide [38]. Since 1994, the population above 60 has doubled in Greenland [6]. We found that the unadjusted incidence rate of IHD, MI, stroke, and AF/AFL increased as expected during the study period, with stroke being the most frequent. Surprisingly, the unadjusted incidence rate of HF showed a decline throughout the study period, aligning with the noticeable decrease in the age- and sex-specific IR of HF. The escalating incidence of CVD in Greenland, particularly among the elderly population, poses a significant challenge for the healthcare system in the future, and the high incidence rate of stroke is alarming and calls for public health efforts to understand the unique risk factors and health care needs in the Greenlandic population to effectively prevent and manage stroke. The healthcare system in Greenland faces challenges in delivering uniformly high-quality healthcare to all citizens. This is largely due to the country's sparse population, which is dispersed across a vast area, with many inhabitants living in remote regions. Adding to these challenges are limited economic resources and difficulties in recruiting and retaining healthcare professionals. The availability of local healthcare facilities and staff varies depending on the location and population size. As a result, these circumstances limit the accessibility and range of options for preventive care. As the proportion of older people with CVD continues to rise, so will the burden on healthcare resources. It is advisable to focus on primary prevention, involving the prevention of the development of modifiable risk factors. Tobacco smoking, overweight/obesity, dyslipidemia, hypertension, hyperglycemia, poor diet, and physical inactivity during childhood are all associated with early onset of atherosclerosis [51]. The prevalence of obesity among preschool children was 21,7 % in 2003 [52], and daily smoking is more common than uncommon in Greenland [22]. In the 2018 public health survey, which included 5.8 % of the population between 15 and 94 years, it was reported that 72 % of smokers expressed a desire to quit, yet only 2 % had concrete plans to do so in the subsequent month [22]. Notably, this desire was most pronounced among younger smokers, with nearly 80 % wishing to stop. The availability of smoking cessation support varied significantly by location, ranging from 9 % to 56 %, with the highest support levels in the capital, Nuuk, and the lowest in eastern Greenland [22]. The survey also revealed that 30 % of individuals with severe obesity (BMI >30) had attempted weight loss in the past six months, compared to 18 % among those classified as obese. Like smoking cessation, the availability of weight loss support was location-dependent, varying from 13 % to 49 %. Additionally, the inclination to alter drinking habits was most common among heavy drinkers, with up to 86 % desiring change [22]. Consequently, a multifaceted effort from society is warranted, with a focus on health education, promoting access to healthy food, encouraging physical activity, and improving access to health care. Research and monitoring the prevalence of CVD, and CVD risk factors in Greenland is crucial for directing intervention strategies and evaluating their effect.

### 4.3 Mortality

Individuals with CVD had more than twice the mortality of those without CVD, highlighting the health implications and potential risk of mortality associated with CVD. Interestingly, men had a 40 % higher mortality risk compared to women, a trend comparable with observations in other populations [53]. The differences in mortality found indicate a need for intensified CVD prevention, especially among men.

### 4.4 Strengths and limitations

This study utilized nationwide registers, which provide a robust representation of the Greenlandic population. Although nationwide and comprehensive, it includes the risk of missing data or underreporting. Evidence of underdiagnosis in Greenland is accumulating. As previously mentioned, the number of unregistered cases of AF/AFL is high, and revascularization rates among Greenlanders are comparable to those of Danes [18,36]. Furthermore, studies indicates that conditions such as osteoporosis and psoriasis are underdiagnosed in the population [54,55]. Hence, we highly suspect that underdiagnosis is prevalent in Greenland, and we expect that the rates found in this study somewhat underestimates the real incidences. Regarding the consistency of underdiagnosis over the study period, opinions differ on whether its incidence has increased or decreased. Consequently, we propose that the variability in underdiagnosis rates could influence the observed changes in disease incidence, although the exact nature of this influence remains uncertain. Further investigation is necessary to understand the impact of underdiagnosis on the trends identified in our research. Our study specifically focused on individuals born in Greenland to Greenlandic-born parents, thereby providing detailed insights into the incidence rates within this population. This approach is particularly relevant considering the significant annual migration into and out of Greenland. Given the limited number of healthcare professionals the possibility of coding errors or misclassifications, can be present based on errors among only a few individuals. Given the population size of Greenland, the number of incident cases is limited, hence a small change in cases per year can have a potentially big influence.

## 5 Conclusion

This study presents insight into CVD trends among the Arctic Inuit population, aligning largely with the observed trends in Western nations. Noteworthy are the elevated IR of stroke and the increasing IR of AF/AFL and MI in men, warranting further investigation. While the age-adjusted IR for most conditions is declining, the unadjusted rates show an increase driven by an aging population, signifying an increasing burden of CVD on the healthcare system. Highlighting the need for proactive preventive measures and initiatives to promote cardiovascular health within the community. The nationwide registers used in this study provide a robust representation of the Greenlandic population. Given the healthcare challenges inherent in Greenland's remote and sparsely populated geography, it is plausible that the actual incidence of CVD may be higher than reported here. This is crucial for understanding CVD in Greenland and for guiding future public health strategies. Therefore, continued research and monitoring of CVD prevalence and risk factors in Greenland are essential to assess the health needs in this population.

## Author contributions

HEL and MEJ conceptualized and designed the study and acquired the data. HEL conducted the statistical analysis, interpreted the data, and drafted the initial manuscript. UWG, FG, MLP and MEJ critically reviewed and revised the manuscript for important intellectual content. All authors approved the final manuscript as submitted and agree to be accountable for all aspects of the work.

## Declaration of competing interest

The authors declare the following financial interests/personal relationships which may be considered as potential competing interests: HEL reports financial support was provided by Karen Elise Jensen Foundation. HEL reports financial support was provided by Novo Nordisk Foundation. HEL reports travel was provided by A. P. Møller Foundation. HEL reports travel was provided by Ketty and Ejvind Lyngsbæks Foundation. FG reports a relationship with Bayer that includes: consulting or advisory. FG reports a relationship with Pfizer that includes: consulting or advisory. FG reports a relationship with Astra-Zeneca that includes: consulting or advisory. FG reports a relationship with Ionis that includes: consulting or advisory. FG reports a relationship with Alnylam that includes: consulting or advisory. FG reports a relationship with Pharmacosmos that includes: consulting or advisory. FG reports a relationship with Abbott that includes: consulting or advisory. FG reports a relationship with Novartis that includes: speaking and lecture fees. FG reports a relationship with Orion Pharma that includes: speaking and lecture fees. MEJ reports a relationship with Astra-Zeneca that includes: funding grants. MEJ reports a relationship with Sanofi Aventis that includes: funding grants. MEJ reports a relationship with Boehringer Ingelheim that includes: funding grants. MEJ reports a relationship with Novo Nordisk that includes: funding grants. MEJ reports a relationship with Novo Nordisk that includes: equity or stocks. If there are other authors, they declare that they have no known competing financial interests or personal relationships that could have appeared to influence the work reported in this paper.
