# Supplementary material for: Trends in cardiovascular disease among Inuit in Greenland from 1994 to 2021
Source: Atheroscler Plus. 2024 Apr 25;56:12–20. doi: 10.1016/j.athplu.2024.04.002 (PMC11112263; doi:10.1016/j.athplu.2024.04.002)
Supplement: Multimedia component 1 [file mmc1.docx]

**Table A***; ICD-10, ICD-8 and ICPC-2 codes according to CVD subgroup*

| **Ischemic Heart Disease:** | |
| --- | --- |
| **ICD-10** | I20.0 – I25.9 |
| **ICD-8** | 410 – 414 |
| **ICPC-2** | K74, K75, K76 |
| **Myocardial infarction:** | |
| **ICD-10** | I21 |
| **ICD-8** | 410 |
| **ICPC-2** | - |
| **Stroke:** | |
| **ICD-10** | I60 – I64, I69.0 – I69.4, G45 |
| **ICD-8** | 430 – 431, 433 – 436 |
| **ICPC-2** | K89, K90 |
| **Heart Failure:** | |
| **ICD-10** | I50.0 – I50.9, I11.0, I13.0, I13.2, I42-I43 |
| **ICD-8** | 426 – 42719 |
| **ICPC-2** | K77 |
| **Atrial Fibrillation / Flutter:** | |
| **ICD-10** | I48 |
| **ICD-8** | 42793 – 42794 |
| **ICPC-2** | K78 |
